# What every intensivist should know about prognostic scoring systems and risk-adjusted mortality

**DOI:** 10.5935/0103-507X.20160052

**Published:** 2016

**Authors:** Mark T. Keegan, Marcio Soares

**Affiliations:** 1 Department of Anesthesiology, Division of Critical Care, Mayo Clinic - Rochester, MN, USA.; 2 Department of Critical Care, Instituto D'Or de Pesquisa e Ensino - Rio de Janeiro (RJ), Brazil.

In the practice of medicine, multiple scores and prognostic systems have been developed to quantify disease severity, assess prognosis, and guide therapeutic interventions. The Glasgow Coma Scale, the Model for End Stage Liver Disease (MELD), and the American Society of Anesthesiologists Physical Status Classification are but a few examples. Heterogeneity in the practice of intensive care medicine, the high cost of care, the very real chance of death in intensive care units (ICUs), and the desire to make comparisons between ICUs have prompted the development and refinement of ICU-specific prognostic systems.^(1-7)^

Scoring systems may be generic or disease-specific, may be used for cohort analysis or individual patient assessment, can be based on physiologic derangement or resource allocation, and may be simple or complex. In critical care practice, two major categories of scoring systems exist. Organ failure scores (e.g., the Sequential Organ Failure Assessment, SOFA) describe a patient's physiologic derangements by organ system to provide an objective assessment of the extent and severity of organ dysfunction. The other major category is the severity-of-illness prognostic model, a discussion of which will occupy the majority of this commentary. These systems (e.g., the Acute Physiology and Chronic Health Evaluation, APACHE) use physiologic data, pre-morbid conditions and information regarding the nature of the current illness to predict the likelihood of mortality.

## Sequential Organ Failure Assessment: an organ dysfunction score

Multiple organ dysfunction syndrome is a major cause of ICU morbidity and mortality. The extent and severity of organ dysfunction may be quantified in a number of organ dysfunction scores, the most prominent of which is the SOFA.^(8)^ Originally designed to be used in patients with sepsis, the SOFA is now used in all patient groups. Daily scores can be calculated and used to track the degree of organ dysfunction throughout a patient's ICU stay - in contrast to generic prognostic systems, which are designed to give a prediction based on the first ICU day alone. Scores between 0 and 4 are assigned to each of the cardiovascular, respiratory, hepatic, hematologic, neurologic and renal systems, depending on the degree of derangement, and are summed to yield a total SOFA score. Such scores were not originally designed to predict mortality, but high absolute scores and an increase in a score within the first 96 hours of ICU care are associated with increased risk of death.^(9)^

## Intensive care unit severity of illness prognostic scoring systems (e.g., APACHE, SAPS, MPM)

There are three major generic ICU prognostic systems: the APACHE, the SAPS (Simplified Acute Physiology Score) and the MPM (Mortality Probability Model).^(10-13)^ After first being described in the 1980s, the models have been updated over time, and their most recent iterations are the APACHE IV, SAPS 3, and MPM III. Table 1 provides details of the most recent versions. The systems use data from acute physiology, acute diagnoses, chronic health conditions, and the characteristics of the index ICU admission to predict hospital mortality. The SAPS and MPM_0_ are calculated from data available within 1 hour of ICU admission and therefore reflect the severity of illness upon admission. The APACHE and MPM_24_ are calculated from data available within the first 24 hours of ICU admission. The APACHE is more complicated - and more accurate - than the SAPS, which in turn is more complicated and more accurate than the MPM.^(14)^ A number of other generic prognostic systems have been developed in specific geographical areas (e.g., Intensive Care National Audit & Research Centre - ICNARC in the United Kingdom and Australian and New Zealand Risk of Death - ANZROD in Australia and New Zealand).^(15,16)^

**Table 1:** Study characteristics, performance and variables of the most recent versions of the most prominent prognostic models

	APACHE IV	SAPS 3	MPM_0_ III
Study population	110,558	16,784	124,855
Study period	January 1, 2002 to December 31, 2003	October 14 to December 15, 2002	October 2001 to March 2004
Geographic regions	USA	35 countries, 5 continents	USA
Number of ICUs	104	303	135
Number of hospitals	45	281	98
Variables in the model	142	20	16
Time of data collection	First 24 hours of ICU admission	± 1 hour of ICU admission	Within 1 h of ICU admission
AUC	0.88	0.848	0.823
HL C statistic	16.9	14.29	11.62 (abs 10.94)
SMR	0.997	1.0	1.018
Predictor variables			
Age	Yes	Yes	Yes
Length of hospital stay before ICU admission	Yes	Yes	No
Type of ICU admission	Yes	Yes	Yes
ICU admission source	8	3	No
Chronic co-morbidities	7	6	3
Resuscitation status	No	No	Yes
CPR before ICU admission	No	No	Yes
Reasons for ICU admission/acute diagnosis	116	10	5
Surgical status at ICU admission	Yes	Yes	No
Anatomical site of surgery	No	5	No
Acute infection at ICU admission	No	Yes	No
Vasoactive drug therapy	Yes	Yes	No
Mechanical ventilation	Yes	Yes	Yes
Clinical physiologic variables	6	4	3
Laboratory physiologic variables	10	6	0

APACHE - Acute Physiology and Chronic Health Evaluation; SAPS - Simplified Acute Physiology Score; MPM - Mortality Probability Model; ICU - intensive care unit; AUC - area under the receiver operating characteristic curve; HL - Hosmer-Lemeshow; SMR - standardized mortality ratio; CPR - cardiopulmonary resuscitation. Source: Afessa B, Gajic O, Keegan MT. Severity of illness and organ failure assessment in adult intensive care units. Crit Care Clin. 2007;23:639-58.

## Methodology for the development of prognostic scoring systems

The outcome of interest, the *dependent* variable, is usually chosen to be mortality - ICU mortality, hospital mortality, or 28-day mortality, for example.^(17,18)^ The binary (or dichotomous) nature of this variable allows the use of logistic regression techniques in model development, although other techniques, including Bayesian analysis, Cox binary regression and neural nets may also be used. Predictor (*independent*) variables, which should be routinely available, exist independently from intervention and be reliable, are chosen based on analytic techniques or (less commonly) expert consensus. Such variables include, for example, age, pH, and the presence or absence of cirrhosis. In a logistic regression analysis, predictor variables (denoted by X) are linked to the probability of death) by a series of coefficients (denoted by β) as follows:

Natural logarithm (ln) of the odds of death = β_0_ + β_1_(X_1_) + β_2_(X_2_) + β_3_(X_3_) + ... β_n_(X_n_)

The odds ratio of death for each unit increase in the variable X_n_ is e^βn^. The coefficients of the logistic function are determined by statistical analyses, with their signs and magnitudes providing indications of the directions and strengths of association, respectively. For the prediction of non-dichotomous variables (e.g., ICU length of stay [LOS]), a linear regression model is used. Inclusion of a greater number of variables in the model usually provides increased predictive accuracy, but at the cost of increased burden of data collection. There should be at least 10 "events" (deaths) for each predictor variable in the model. Ideally, a highly accurate but parsimonious model with few predictor variables is desired. Models that have been developed on large multi-institutional databases and validated on separate datasets are preferred.

## Assessment of model performance

Model performance should be assessed through the evaluation of discrimination and calibration. *Discrimination* quantifies the accuracy of a given prediction. For example, if a prognostic system predicts a mortality of 80% for a cohort of patients with a certain APACHE IV score, discrimination is perfect if 80% of that group of patients die. The area under a receiver operating characteristic curve (AUC) is used as a measure of discrimination.^(19)^ Perfect discrimination will give rise to an AUC of 1; an AUC of 0.5 signifies that the model prediction is no better than chance.

**Figure 1:**
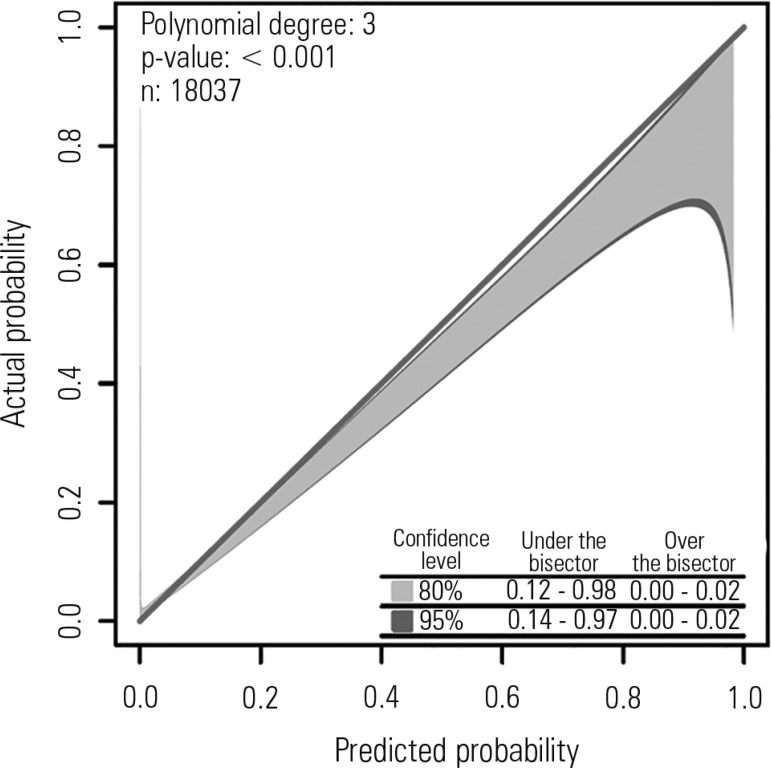
Calibration belt for the Simplified Acute Physiology Score 3. Standardized mortality ratios with their 95% confidence intervals. The degree of the polynomial, the Wald statistics results and the number of patients are given in the upper-left quadrant. In the lower right quadrant, the times the calibration belt significantly deviates from the bisector using 80% (light gray area) and 95% (dark gray boundaries) confidence levels are reported. Courtesy: Dr. Pedro Brasil.

Calibration is a measure of how well a particular model performs over a wide range of predicted mortalities. It is evaluated by the Hosmer-Lemeshow (HL) goodness of fit statistic, which is calculated by grouping a cohort of patients into deciles of predicted risk and comparing the observed to predicted mortalities across deciles to give a chi-square statistic. A p-value greater than 0.05 (i.e., non-significant) implies good calibration. The HL statistic is affected by sample size.^(20)^ Some investigators have proposed the calibration belt as an alternative method to assess a model's calibration.^(21)^ The calibration belt uses a generalized polynomial logistic function between the outcome and the logit transformation of the estimated probability, providing information on the direction, extent, and risk classes affected by deviations between the observed and predicted mortality (Figure 1).

Accuracy refers to the difference between predictions and observed outcomes at the level of individual patients and may be measured by the Brier score, which measures the average squared deviation between predicted probabilities for a set of events and their outcomes.

## Customization

Changes in clinical practice and in case mix lead to the deterioration of prognostic performance over time.^(22)^ In addition, models may perform suboptimally in certain geographic regions or patient populations. Customization is the process by which a model is modified to improve its accuracy, either by altering the coefficients in the equation (1^st^-level customization) or changing the variables (2^nd^-level customization). For example, the original description of SAPS 3 provided customized equations for different geographic regions (e.g., South America, Eastern Europe) to optimize its performance in those regions. The ability to compare different ICUs, institutions, or countries is compromised if customized models are used in each location because customization limits a model's external validity. Nonetheless, the technique is an attractive alternative to the onerous process of developing and validating a new prognostic model.

## Standardized ratios

The ratio of observed mortality to the prognostic scoring system-predicted mortality of a cohort of patients is the standardized mortality ratio (SMR), and it should be reported with a 95% confidence interval. The SMR is widely used to evaluate performance because mortality is the most objective outcome measure and is not prone to error. If the 95% CI of the SMR includes 1, the performance of the institution or unit is considered average. If the 95% CI does not include 1, SMRs less than 1 and greater than 1 are considered indicative of good and poor performances, respectively. However, the SMR is not a perfect measure; in addition to differences in the quality of care, it may be influenced by the accuracy of the prognostic model, artifacts of data collection or analysis, case mix, lead-time bias, and inter-rater reliability. Other outcome measures (e.g., duration of mechanical ventilation, ICU LOS) are also suitable for the calculation of observed-to-predicted ratios. Specifically for resource use (using ICU LOS as a proxy), some investigators have used a different approach to assess the standardized severity-adjusted resource use (SRU) for each individual ICU. In this case, SRU estimates the average amount of resources used per surviving patient in a specific ICU.^(23)^

## Uses of prognostic scoring systems

Mortality rates, adjusted based on the predictions of mortality provided by prognostic scoring systems, are increasingly used to compare the quality of care provided by different ICUs and hospitals. These "severity-adjusted mortality rates" can be used for "*benchmarking*" against similar institutions or institutions recognized to be high-performing to identify institutional deficiencies in clinical outcome and highlight areas for improvement. Third-party payors may use severity-adjusted mortality rates as one criterion for choosing health care providers, and performance data can facilitate the accreditation process by external agencies. Within the same organization, comparisons of care among different ICUs can be made, and a single unit's performance over time may be evaluated to highlight evolving changes in the quality of care. Prognostic systems may serve as tools for evaluating the impact of new therapies or organizational changes as part of *quality improvement* initiatives. From a *resource use* standpoint, such systems may help to identify a cohort of ICU patients with low mortality risk who could be managed in a non-ICU setting, such as a progressive care ("step down") unit and may also assist with end-of-life decision making.^(24,25)^ Prognostic models may help answer ICU outcomes research questions and may aid with risk stratification of patients for entry into clinical trials, although this latter approach is controversial because of calculation complexity, timing, and inter-observer variability.^(26)^ Completed trials may be subject to post-hoc analyses using risk stratification of subgroups, leading to the generation of further hypotheses.

## Limitations to the use of prognostic models for clinical decision support

Although there are numerous examples of the use of prognostic models to make decisions for individual patients (e.g., use of the Model for End-Stage Liver Disease [MELD] score for organ allocation for liver transplantation), such use is not without problems.^(27)^ Prognostic scoring systems perform best at a cohort level. For example, in a cohort of 1000 patients with a predicted mortality of 90%, 100 patients will, on average, survive - despite a predicted mortality of 90% for any individual patient. These 100 patients will have confirmed, rather than undermined, the validity of the model. In addition to the inherent uncertainty concerning prediction in individual patients, even the best clinically useful models have AUCs no higher than 0.9, implying imperfection even for cohort outcome prediction. Furthermore, model performance may be hampered by the non-availability of all data required for score calculation - missing data are counted as normal - and by errors in collecting and entering data, as well as patient preferences for life-support.^(14,28)^ Barriers to widespread acceptance of prognostic models include the cost of the information technology infrastructure required to acquire data for complex models, clinician resistance because of perceived superiority of their own estimates of patient survival or their disregard for the model's relevance for their patients, and the focus on prediction of mortality rather than functional outcome, such as quality of life years.

## The future

Updated versions of the major prognostic systems are expected and will be welcome. Of potentially more use, however, will be innovative models that may be derived through advances made possible by the era of "big data", including "machine learning" algorithms and dynamic reassessments of outcome predictions.^(29,30)^ Widespread implementation of electronic medical records, coupled with techniques of big data analytics pioneered in the retail and banking industries, may ultimately allow reliable, well-presented, patient-level prediction of functional outcomes. Furthermore, we may be close to a scenario wherein clinicians will trust such predictions and accept computer-generated risk mitigation or "course correction" strategies.
